# Indomethacin augments lipopolysaccharide-induced expression of inflammatory molecules in the mouse brain

**DOI:** 10.7717/peerj.10391

**Published:** 2020-11-18

**Authors:** Mona Yasin Mohamed, Willias Masocha

**Affiliations:** Department of Pharmacology and Therapeutics, Faculty of Pharmacy, Kuwait University, Safat, Kuwait

**Keywords:** Lipopolysaccharide, Neuroinflammation, Proinflammatory cytokines, Inducible nitric oxide synthase, Indomethacin, Gene expression, Brain, Minocycline, Anti-inflammatory cytokine

## Abstract

Indomethacin and other non-steroidal anti-inflammatory drugs (NSAIDs) are used to relieve pain and fever including during infections. However, some studies suggest that NSAIDs protect against neuroinflammation, while some find no effects or worsening of neuroinflammation. We evaluated the effect of indomethacin alone on in combination with minocycline, a drug that inhibits neuroinflammation, on the expression of transcripts of neuroinflammatory molecules-induced by lipopolysaccharide (LPS) in the brain of mice. Inoculation of male BALB/c mice with LPS induced the expression of the microglia marker ionized calcium binding adaptor molecule protein, mRNA expression of the genes for cytokines interleukin-1beta (*Il1b*) and tumor necrosis factor-alpha (*Tnf*) and inducible nitric oxide synthase gene (*Nos2*), but not *Il10*, in the brain. Treatment with indomethacin had no significant effect on the cytokines or *Nos2* mRNA expression in naïve animals. However, pretreatment with indomethacin increased LPS-induced *Nos2* mRNA and inducible nitric oxide (iNOS) protein expression, but had no significant effect on LPS-induced mRNA expression of the cytokines. Minocycline reduced LPS-induced *Il1b* and *Tnf*, but not *Nos2*, mRNA expression. Treatment with indomethacin plus minocycline had no effect on LPS-induced *Il1b, Tnf* and *Nos2* mRNA expression. In conclusion these results show that indomethacin significantly augments LPS-induced *Nos2* mRNA and iNOS protein expression in the brain. In the presence of indomethacin, minocycline could not inhibit LPS-induced pro-inflammatory cytokine expression. Thus, indomethacin could exacerbate neuroinflammation by increasing the expression of iNOS and also block the anti-inflammatory effects of minocycline.

## Introduction

Neuroinflammation is one of the features shared by a lot of neurodegenerative diseases and infectious diseases (Chen, Zhang & Huang, 2016; Guo & Buch, 2019; Masocha & Parvathy, 2009; Meneses et al., 2019; Molteni & Rossetti, 2017; Schain & Kreisl, 2017). Some of its features include microglia activation. Activated microglia undergo morphological changes into amoeboid form accompanied with changes in their gene expression pattern. Activated microglia increase the expression of different mediators such as tumor necrosis factor (TNF-α), interleukin-1 beta (IL-1β), IL-12, reactive oxygen species (ROS), nitric oxide (NO), prostaglandins and chemokines, which start a cascade of immune reaction to the site of inflammation aimed at destroying the invading pathogen or dead cell but can also cause neuronal damage (Loane & Byrnes, 2010; Lull & Block, 2010; Wolf, Boddeke & Kettenmann, 2017).

Microglia can be activated by several pro-inflammatory stimuli such as lipopolysaccharide (LPS), an endotoxin obtained from the Gram-negative bacteria’s outer membrane surface. Lipopolysaccharide induces cyclooxygenase-2 (COX-2), inducible nitric oxide synthase (iNOS) and pro-inflammatory cytokine expression in the brain (Biesmans et al., 2013; Catorce & Gevorkian, 2016; Edan, Luqmani & Masocha, 2013; Qin et al., 2007). Treating cell cultures or animals with LPS has been used to activate microglia and to study the effect of anti-inflammatory drugs on neuroinflammation (Edan, Luqmani & Masocha, 2013; Fan et al., 2005; Henry et al., 2008; Hoogland et al., 2015; Kim et al., 2004).

Indomethacin, a non-steroidal anti-inflammatory drug (NSAID), is mainly used for treatment of pain, inflammation, and fever in several chronic inflammatory diseases (Eccleston et al., 2017; Nalamachu & Wortmann, 2014). In terms of neuroinflammation, indomethacin and other NSAIDs have been reported to have different effects on LPS-induced microglia activation and pro-inflammatory molecule expression; that is, inhibition, no effect and augmentation of LPS-induced neuroinflammatory effects (Aid, Langenbach & Bosetti, 2008; Banks et al., 2015; Blais, Turrin & Rivest, 2005; Na et al., 2015; Nakamori et al., 1994; Sacco et al., 1998; Teeling et al., 2007).

In this study, we evaluated the LPS-induced neuroinflammation in the brain by measuring the expression of the microglia marker ionized calcium binding adaptor molecule (Iba-1) protein, the transcripts of the genes of pro-inflammatory molecules that is, the cytokines interleukin 10 (*Il10*), *Il1b* and tumor necrosis factor-alpha (T*nf*) and as well as the inducible nitric oxide synthase 2 (*Nos2*). We evaluated the effects of pretreatment with indomethacin on LPS-induced expression of the transcripts of *Il10, Il1b, Tnf* and *Nos2*, as well as the iNOS protein. We also evaluated the effects of pretreatment with indomethacin plus minocycline, a drug that inhibits neuroinflammation (Guo & Bhat, 2007; Henry et al., 2008; Hou et al., 2016; Plane et al., 2010; Stirling et al., 2005; Tikka et al., 2002; Yrjänheikki et al., 1998), on LPS-induced expression of the transcripts of *Il1b, Tnf* and *Nos2*.

## Materials and Methods

### Animals, LPS administration and drug treatment

One hundred and ten male BALB/c mice (8–12 weeks old; 20–30 g) provided by the breeding unit at the Health Science Center, Kuwait University were used in this study. Animals were kept in temperature controlled (24 ± 1 °C) rooms on a 12-h light/dark cycle and constantly checked for signs of disease. All animals were provided with standard food and water ad libitum. All animal experiments were approved by the Ethical Committee for use of Laboratory Animals in Teaching and Research of the Health Sciences of Kuwait University.

All the drugs were administered at a volume of 10 ml/kg body mass.

The first group of mice (*n* = 16) either received a single intraperitoneal (i.p.) dose of LPS from *Escherichia coli* (strain O111:B4, Sigma–Aldrich, St. Louis, MO, USA, *n* = 8) 1 mg/kg or its solvent (phosphate buffered saline, PBS, *n* = 8). The dose of LPS was selected based on those previously shown to induce microglia activation and cytokine expression (Edan, Luqmani & Masocha, 2013).

In the second group (*n* = 8), mice were either treated daily for 3 days with indomethacin 10 mg/kg (Sigma–Aldrich, St. Louis, MO, USA, *n* = 4) dissolved in peanut oil or with its vehicle (*n* = 4), and sacrificed 4 h after the last dose of indomethacin. The dose of indomethacin used in this study is based on that reported to alleviate LPS-induced arthritis (Masocha & Parvathy, 2009).

In the third group, 30 mice were divided into three treatment groups (untreated control, vehicle plus LPS, and indomethacin plus LPS, *n* = 10 per group) and treated i.p. daily for 3 days with indomethacin 10 mg/kg (Sigma–Aldrich, St. Louis, MO, USA) or its vehicle, commencing 2 days before LPS inoculation. On the third day after drug treatment the mice were inoculated with either LPS or its vehicle and sacrificed after 4 h.

In the fourth group, 24 mice were divided into three treatment groups (untreated control, vehicle plus LPS, and minocycline plus LPS, *n* = 8 per group) and treated i.p. daily for 3 days with minocycline 50 mg/kg (Sigma–Aldrich, St. Louis, MO, USA) dissolved in PBS or its vehicle, commencing 2 days before LPS inoculation. On the third day after drug treatment the mice were inoculated with either LPS or its vehicle and sacrificed after 4 h. The dose of minocycline used in this study is based on that reported to inhibit LPS-induced microglia activation (Henry et al., 2008).

In the fifth group, 32 mice were divided into three treatment groups (untreated control (*n* = 9), vehicle plus LPS (*n* = 9), and indomethacin plus minocycline plus LPS (*n* = 14)) and treated i.p. daily for 3 days with indomethacin plus minocycline or their vehicles, commencing 2 days before LPS inoculation. On the third day after drug treatment the mice were inoculated with either LPS or its vehicle and sacrificed after 4 h.

On the last day of drug treatment and LPS inoculation mice were deeply anaesthetized with halothane, sacrificed by decapitation, brains removed and dissected out, snap frozen in liquid nitrogen and kept at −70 °C until protein or mRNA extraction.

### Wes™ capillary-based protein electrophoresis

The protein expression of Iba-1 and iNOS was measured using the automated Wes™ capillary-based protein electrophoresis (ProteinSimple, San Jose, CA, USA) and the 12–230 kDa separation module following the manufacturer’s user guide as described previously (Aly, Khajah & Masocha, 2020). Briefly, half of the fresh frozen brains were homogenized, and protein concentrations determined as described previously (Aly, Khajah & Masocha, 2020). Brain homogenates were diluted to 1 mg/mL using 0.1X buffer. Four parts of the sample was mixed with one part of 5X fluorescent mix, followed by denaturation at 95 °C for 5 min. The primary antibodies for rabbit anti-Iba-1 (Boster Bio, Pleasanton, CA, USA), mouse monoclonal anti-iNOS (OriGene Technologies, Rockville, MD, USA), and rabbit anti-β-actin (Cell Signaling, Danvers, MA, USA; housekeeping protein) were diluted in a ratio of 1:50, using the antibody diluent. The secondary anti-rabbit and anti-mouse antibodies were supplied with the kits (ProteinSimple, San Jose, CA, USA). The assay plate was loaded following the manufacturer’s user guide and centrifuged at 2,500 rpm for 5 min at room temperature. The plate and the capillaries were loaded onto the automated Wes instrument for electrophoresis and immunodetection. At the end of the run data generated were analyzed using the Compass for Simple Western software (ProteinSimple, San Jose, CA, USA). The ratio between of the areas of the resulting electropherograms for Iba-1 and β-actin were calculated (as shown in Fig. S1).

### Real time RT-PCR

Expression of *Il10*, *Il1b*, *Tnf* and *Nos2* mRNA was quantified relative to the expression of the house keeping gene *Ppia* (cyclophilin A) using real time PCR. Total RNA was extracted from half of the fresh frozen brains, reverse-transcribed into cDNA and real time PCR performed on an ABI Prism^®^ 7500 sequence detection system (Applied Biosystems) as described previously (Masocha, 2009). The primers for *Ppia, Il10, Il1b, Tnf* and *Nos2* were purchased from Invitrogen Life Technologies. The primer sequences are listed in Table 1. The threshold cycle (Ct) values for all cDNA samples were obtained and the level of mRNA for each sample were normalized to *Ppia* (housekeeping gene) ΔCt. The relative expression of the gene of interest was calculated using −2^−ΔΔCt^ method using the following equations (Livak & Schmittgen, 2001).

**Table 1:** PCR primer sequences of cyclophilin, cytokines and nitric oxide synthase 2.

Gene	Polarity	Sequence 5′–3′	GenBank*
*Ppia* (cyclophilin A)	Sense Anti-sense	*GCTTTTCGCCGCTTGCT* *CTCGTCATCGGCCGTGAT*	X52803
*Tnf* (tumor necrosis factor alpha)	Sense Anti-sense	*GGCTGCCCCGACTACGT* *GACTTTCTCCTGGTATGAGATAGCAAA*	NM-013693
*Il10* (interleukin 10)	Sense Anti-sense	*CAGCCGGGAAGACAATAACTG* *CCGCAGCTCTAGGAGCATGT*	NM-010548
*Il1b* (interleukin 1 beta)	Sense Anti-sense	*TGGTGTGTGACGTTCCCATT* *CAGCACGAGGCTTTTTTGTTG*	NM-008361
*Nos2* (nitric oxide synthase 2)	Sense Anti-sense	*AACATCAGGTCGGCCATCA* *CGTACCGGATGAGCTGTTGAA*	M84373

**Note:**

* GenBank accession numbers.

### Statistical analyses

Data were tested for normality using the D’Agostino-Pearson normality test; if data passed the normality test, that is, were normally distributed, parametric tests were used, however, if they failed the normality test, that is, were not normally distributed, non-parametric tests were used. Further statistical analysis was performed using Student’s *t* test, one-way analysis of variance (ANOVA) followed by Tukey multiple comparison test for normally distributed data and Mann–Whitney *U* test or Kruskal–Wallis test followed by Dunn’s multiple comparison test for non-normal data using GraphPad Prism software (version 6.0). The differences were considered significant at *p* < 0.05. The results in the text and figures are expressed either as the mean ± SEM or a median and interquartile range.

## Results

### Effect of LPS on expression of proinflammatory and anti-inflammatory molecules in the brain

Lipopolysaccharide induced the protein expression of the microglia marker Iba-1 and increased mRNA of the proinflammatory molecules *Il1b, Tnf* and *Nos2*, but had no effect on the mRNA of the anti-inflammatory cytokine *Il10*, in the brains of mice compared to vehicle treatment (Figs. S2 and S3).

### Effect of indomethacin on *Il10, Il1b, Tnf* and *Nos2* mRNA expression

**Figure 1:**
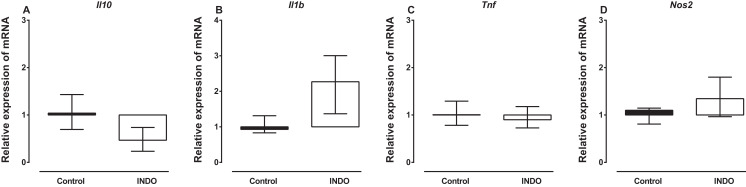
Effect of treatment of BALB/c mice with indomethacin on *Il10*, *Il1b*, *Tnf* and *Nos2* mRNA expression. Relative expression of (A) *Il10*, (B) *Il1b*, (C) *Tnf* and (D) *Nos2* mRNA in the brain of control (vehicle-only) and indomethacin-treated mice. Each box and whiskers represent the median and interquartile range of the values obtained from four animals. There was no statistically significant difference with vehicle-treated control animals (Mann–Whitney *U* test).

Treatment with indomethacin had no significant effect on *Il10*, *Il1b, Tnf* and *Nos2* mRNA in the brains of mice compared to vehicle treatment. The Mann–Whitney *U* test showed there was no difference in the mRNA of *Il10*, *Il1b, Tnf* and *Nos2* between the brains of mice treated with indomethacin compared to vehicle-treated control mice (*U* = 2, *p* = 0. 0.1143; *U* = 1, *p* = 0.0571; *U* = 5, *p* = 0.4857; *U* = 3, *p* = 0. 0.4857, respectively; Fig. 1). However, there was a trend to increase the mRNA of *Il1b* (median of relative expression 2.269) and to decrease the mRNA of *Il10* (median of relative expression 0.4671) in the brains of mice treated with indomethacin compared to vehicle-treated control mice.

### Effects of treatment with indomethacin on LPS-induced changes of *Il10, Il1b, Tnf* and *Nos2* mRNA expression

**Figure 2:**
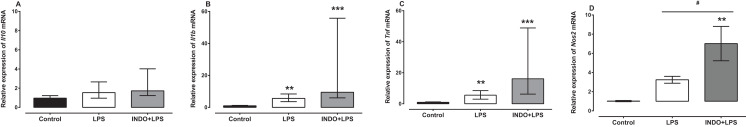
Effect of treatment with indomethacin on LPS-induced changes of *Il10*, *Il1b*, *Tnf* and *Nos2* mRNA expression. Relative expression of (A) *Il10*, (B) *Il1b*, (C) *Tnf* and (D) *Nos2* mRNA in the brains of uninoculated control, LPS-inoculated vehicle-treated and LPS-inoculated indomethacin-treated mice at 4 h post inoculation. Each bar or box and whiskers represents the mean ± the SEM (C) or median and interquartile range (A and B), respectively, of the values obtained from ten animals. **p < 0.01, ***p < 0.001 compared to vehicle-treated control, and #*p* < 0.05 compared to mice inoculated with LPS (one-way ANOVA followed by Tukey multiple comparison test for *Nos2* or Kruskal–Wallis test followed by Dunn’s multiple comparison test for *Il10*, *Il1b* and *Tnf*).

Treatment with indomethacin had no significant effect on LPS-induced *Il1b* and *Tnf* mRNA, but significantly increased *Nos2* mRNA (Fig. 2). For *l10* mRNA, a Kruskal–Wallis test showed that there was no statistically significant difference in transcript levels between the treatment groups (*H*(2) = 4.74, *p* = 0.0921; Fig. 2A). For *l1b* and *Tnf* mRNA, a Kruskal–Wallis test showed that there was a statistically significant difference in transcript levels between the treatment groups (*H*(2) = 21.24, *p* < 0.0001; *H*(2) = 22.17, *p* < 0.0001, respectively). However, a Dunn post-hoc test showed that there was no difference in the relative expression of *Il1b* and *Tnf* mRNA between the brains of LPS-inoculated mice treated with indomethacin and those of LPS-inoculated mice treated with vehicle (*p* > 0.05; Figs. 2B and 2C). For *Nos2* mRNA there was a statistically significant difference between the treatment groups as determined by one-way ANOVA (*F*(2, 27) = 8.247, *p* = 0.0016). A Tukey post-hoc test showed that the brain of LPS-inoculated mice treated with indomethacin had significantly higher relative expression of *Nos2* mRNA compared to of LPS-inoculated mice treated with vehicle (*p* < 0.05; Fig. 2D).

### Effects of treatment with indomethacin on LPS-induced changes of iNOS protein expression

**Figure 3:**
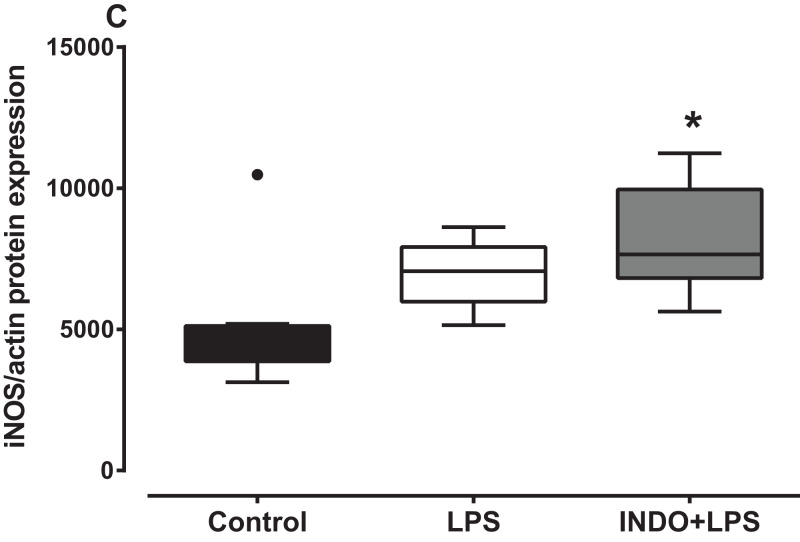
Effect of treatment with indomethacin followed by LPS-induced inoculation on iNOS protein expression. Protein expression of iNOS in the brains of uninoculated control, LPS-inoculated vehicle-treated and LPS-inoculated indomethacin-treated mice at 4 h post inoculation. Each box and whiskers represents the median and interquartile range of the values obtained from six to eight animals. The dot above the control shows an outlier value. *p < 0.05 compared to vehicle-treated control (Kruskal–Wallis test followed by Dunn’s multiple comparison test).

There was a significant increase in iNOS protein expression in the brain of LPS-inoculated mice treated with indomethacin, but not vehicle, compared to uninoculated control mice (Fig. 3). There was a statistically significant difference in iNOS protein levels between the treatment groups as determined by Kruskal–Wallis test (*H*(2) = 8.778, *p* = 0.0075). A Dunn post-hoc test showed that the brain of LPS-inoculated mice treated with indomethacin had significantly higher relative expression of iNOS protein compared to the brain of uninoculated mice treated with vehicle, but not LPS-inoculated mice treated with vehicle (*p* < 0.05; *p* > 0.05, respectively; Fig. 3).

### Effects of treatment with minocycline alone or with indomethacin on LPS-induced changes of *Il1b, Tnf* and *Nos2* mRNA expression

**Figure 4:**
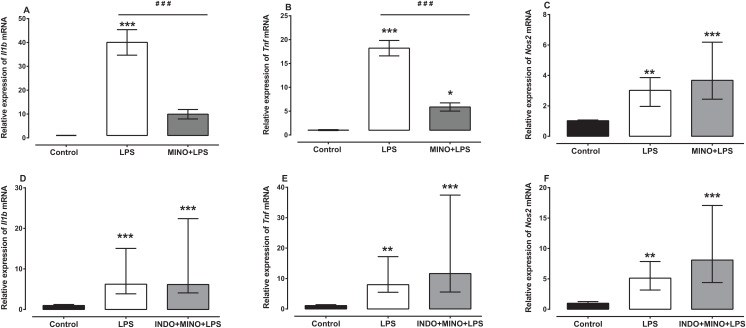
Effect of treatment with minocycline or co-treatment with indomethacin plus minocycline on LPS-induced upregulation of *Il1b*, *Tnf* and *Nos2* mRNA expression. Relative expression of (A) *Il1b*, (B) *Tnf* and (C) *Nos2* mRNA in the brains of uninoculated control, LPS-inoculated vehicle-treated and LPS-inoculated minocycline-treated mice at 4 h post inoculation. Each bar represents the mean ± the SEM (A and B) or median and interquartile range (C) of the values obtained from eight animals. Relative expression of (D) *Il1b*, (E) *Tnf* and (F) *Nos2* mRNA in the brains of uninoculated control, LPS-inoculated vehicle-treated and LPS-inoculated indomethacin plus minocycline-treated mice at 4 h post inoculation. Each bar represents the median and interquartile range of the values obtained from nine to fourteen animals. *p < 0.05, **p < 0.01, ***p < 0.001 compared to vehicle-treated control, and ###*p* < 0.001 compared to mice inoculated with LPS (one-way ANOVA followed by Tukey multiple comparison test for (A) and (B) or Kruskal–Wallis test followed by Dunn’s multiple comparison test for (C–F).

Treatment with minocycline markedly reduced LPS-induced *Il1b* and *Tnf mRNA*, but not *Nos2* mRNA (Figs. 4A–4C). For *Il1b* and *Tnf* mRNA there was a statistically significant difference between the treatment groups as determined by one-way ANOVA (*F*(2, 21) = 38.36, *F*(2, 21) = 69.77, respectively, *p* < 0.0001). A Tukey post-hoc test showed that the brain of LPS-inoculated mice treated with minocycline had significantly lower relative expression of *Il1b* and *Tnf* mRNA compared to LPS-inoculated mice treated with vehicle (*p* < 0.001; Figs. 4A and 4B). For *Nos2* mRNA, a Kruskal–Wallis test showed that there was a statistically significant difference in transcript levels between the treatment groups (*H*(2) = 15.61, *p* = 0.0004). However, a Dunn post-hoc test showed that there was no difference in the relative expression of *Nos2* mRNA between the brains of LPS-inoculated mice treated with minocycline and those of LPS-inoculated mice treated with vehicle (*p* > 0.05; Fig. 4C).

Co-administration of indomethacin with minocycline had no significant effect on LPS-induced *Il1b, Tnf* and *Nos2* mRNA (Figs. 4D–4F). A Kruskal–Wallis test showed that there was a statistically significant difference in *Il1b, Tnf* and *Nos2* mRNA expression between the treatment groups (*H*(2) = 18.82, *p* < 0.0001; *H*(2) = 19.03, *p* < 0.0001; *H*(2) = 18.18, *p* < 0.0001, respectively). However, a Dunn post-hoc test showed that there was no difference in the relative expression of *Il1b, Tnf* and *Nos2* mRNA between the brains of LPS-inoculated mice co-treated with indomethacin plus minocycline and those of LPS-inoculated mice treated with vehicle (*p* > 0.05; Figs. 4D–4F).

## Discussion

Systemic administration of LPS has been shown activate microglia and induce neuroinflammation including induction of the expression of the proinflammatory cytokines, *Nos2* and iNOS (Czapski et al., 2007; Edan, Luqmani & Masocha, 2013; Hom et al., 1995; Jacobs et al., 1997; Qin et al., 2007). Therefore, bacterial LPS has been used in models studying neuroinflammation and evaluating drugs that can inhibit neuroinflammation (Edan, Luqmani & Masocha, 2013; Henry et al., 2008; Iliev et al., 2001; Marchalant, Rosi & Wenk, 2007). In the current study, LPS increased the protein expression of the microglia marker Iba-1 and transcript levels of *Nos2* and the proinflammatory cytokines *Il1b* and *Tnf*, but had no effect on the transcript levels of the anti-inflammatory cytokine *Il10*, in the brain. Treatment with indomethacin had no significant effects in the transcripts of the cytokines *Il10, Il1b* and *Tnf* and *Nos2* transcripts in the brains of naïve animals. Treatment with indomethacin enhanced the LPS-induced elevation in the transcripts of *Nos2*, but not the cytokines *Il10, Il1b* and *Tnf* transcripts. Indomethacin also caused an increased expression of iNOS in the brains of LPS-inoculated mice, whereas LPS on its own did not, compared to uninoculated vehicle-treated mice brains. Treatment with minocycline inhibited the LPS-induced elevation in the transcripts of the cytokines *Il1b* and *Tnf*, but not *Nos2* transcripts. However, in the presence of indomethacin, minocycline lost its inhibitory effects on LPS-induced elevation in the transcripts of the cytokines that is, co-treatment with indomethacin plus minocycline had no effect on the LPS-induced elevation in the transcripts of the cytokines (*Il1b* and *Tnf*) and *Nos2*.

Microglia are a well-known source of proinflammatory cytokines (Conti et al., 2020; Giulian & Baker, 1986; Sawada et al., 1989; Smith et al., 2012; Xu, He & Bai, 2016). Based on that, the upregulation of Iba-1 protein expression as an indicator of microglia activation (Huang et al., 2008; Ito et al., 1998; Ito et al., 2001) in the brains of LPS-inoculated animal was investigated. Iba-1 protein was upregulated in the brain after LPS inoculation, which confirmed the activation of microglia, similar to what has been reported in other studies using western blot or immunohistochemistry techniques (Hoogland et al., 2015; Hu et al., 2011; Reinert et al., 2014).

Inoculation with LPS increased *Tnf, Il1b* and *Nos2* mRNA, but did not affect *Il10* mRNA in the brain of BALB/c mice. These findings of LPS-induced increase in the levels of proinflammatory cytokine transcripts (*Tnf and Il1b*) agree with several previous studies (Edan, Luqmani & Masocha, 2013; Layé et al., 1994; Murray, Skelly & Cunningham, 2011; Qin et al., 2007). In the current study LPS did not alter the expression of IL-10 transcripts similar to a previous study (Szot et al., 2017). However, some studies have shown that LPS increases IL-10 transcripts in the brain (Yang et al., 2020; Neupane et al., 2018). The findings of LPS-induced increase in the levels of *Nos2* transcripts in the brain are also in agreement with previous studies (Murray, Skelly & Cunningham, 2011; Voronova et al., 2016). Thus, the current data correlate with the previous reported findings that LPS-induced proinflammatory cytokines and *Nos2* expression, which elicit an inflammatory response in the brain.

Currently, NSAIDs, which produce their effects via inhibition of COX activity and consequently prostaglandin (PG) synthesis, are one of the main drugs used to relieve pain in many chronic inflammatory diseases, such as rheumatoid arthritis (Hussain et al., 2012). However, the exact role of COX inhibition in systemic inflammation caused by LPS and their effect on neuroinflammation remains controversial. A previous study showed that the NSAID indomethacin can reverse LPS-induced behavioral changes including sickness without changing peripheral or central increase of *Il1b* and *Tnf* mRNA levels (Teeling et al., 2007). However, another study showed that COX-2–deficient mice showed a higher increase in *Nos2*, *Tnf* and *Il1b* gene expression and iNOS, TNF-α and IL-1β protein levels after LPS inoculation when compared to normal mice, which increased their susceptibility to LPS-induced damage (Aid, Langenbach & Bosetti, 2008). This study by Aid, Langenbach & Bosetti (2008) and our study agree with another study that showed that indomethacin, a COX inhibitor, increased the inflammatory response in the brain after systemic LPS administration (Blais, Turrin & Rivest, 2005). On the other hand, the current study revealed that indomethacin had no significant effect on LPS-induced elevation in cytokine transcripts, similar to what was reported previously (Teeling et al., 2007). Previous studies have reported different effects of indomethacin on LPS-induced increase in IL-1 β and TNF-α protein in the brain that is, in some studies it had no effects on LPS-induced increase in IL-1 β and TNF-α protein in the brain (Nakamori et al., 1994; Sacco et al., 1998), whereas in another study it inhibited the LPS-induced increase in IL-1 β protein but had no effect on TNF-α protein in the brain (Banks et al., 2015). In the current study, indomethacin significantly increased the LPS-induced elevation in *Nos2* transcripts and pretreatment with indomethacin before LPS increased iNOS protein levels when LPS alone did not, which is agreement to the study by Aid et al that COX-2 inhibition or deletion increases *Nos2* transcripts and iNOS protein expression when LPS is inoculated (Aid, Langenbach & Bosetti, 2008). On the other hand, various previous studies have reported that indomethacin inhibits LPS-induced increase in *Nos2* transcripts and iNOS protein in macrophages cell culture and rat cerebellum (Aeberhard et al., 1995; Di Girolamo et al., 2003; Pang & Hoult, 1996). The difference between the effects of indomethacin on LPS-induced elevation in *Nos2* transcripts in the current study and that of DiGirolamo and colleagues (Di Girolamo et al., 2003) could be due the difference in animal species used that is, mice versus rats or the number of doses that is, three doses over three days compared to a single dose. Repeated or chronic administration of indomethacin has been shown to cause microglia activation or enhance LPS-induced inflammation (Na et al., 2015; Prechel et al., 2000). Various studies investigating the role of NSAIDs in treating neurodegenerative diseases have produced conflicting results. In a randomized clinical trial, indomethacin was shown to protect Alzheimer’s disease (AD) patients by having a slower degree of cognitive decline when compared to those on placebo (Rogers et al., 1993). However, the positive result is difficult to interpret because 42% of the patients abandoned the study due to adverse events. Another study, a double blinded clinical trial, showed that indomethacin did not slow the progression of AD (De Jong et al., 2008). The findings of the current study show that indomethacin, or inhibition of COX enzymes, is proinflammatory rather that anti-inflammatory in the CNS during LPS-induced neuroinflammation.

Minocycline significantly reduced LPS-induced elevation in the transcripts of *Tnf* and *Il1b*, similar to what has been reported previously (Henry et al., 2008; Hou et al., 2016; Tomás-Camardiel et al., 2004). These findings support the notion of that minocycline has anti-inflammatory effect and ameliorates neuroinflammation. However, minocycline had no effect on LPS-induced increase of *Nos2* mRNA expression in the brain, similar to what reported previously (Tomás-Camardiel et al., 2004). This is contrary to previous in vitro studies, which showed that minocycline inhibited LPS-induced elevation of *Nos2* mRNA and NO production in cultured retinal microglial cells (Lee et al., 2004). The discrepancy could be due to the fact in vitro there were only microglia cells, whereas in vivo there are other cells and molecules that can influence the effect of minocycline.

In the current study, co-treatment with indomethacin plus minocycline had no significant effect on the LPS-induced elevation in *Il1b* and *Tnf* transcripts. These results suggest that, the beneficial effect of minocycline in reducing the LPS-induced expression of the proinflammatory cytokines *Tnf* and *Il1b* transcripts found in this study were annulled by indomethacin. Co-treatment with indomethacin plus minocycline also had no significant effect on LPS-induced elevated levels of *Nos2* transcripts. These results suggest that minocycline protected against the indomethacin-induced worsening of LPS effects. The results of the current study suggest that although minocycline blocks indomethacin augmentation of LPS-induced elevation in *Nos2* transcripts, it loses its beneficial effects of reducing LPS-induced proinflammatory cytokine expression.

## Conclusions

In conclusion, the results of this study show that indomethacin augments the LPS-induced elevation in *Nos2* transcripts levels in the brain, induces elevation of iNOS proteins in combination with LPS, whereas LPS on its own did not. However, indomethacin had no significant effects on cytokine transcripts on its own or on LPS-induced elevation in transcripts of proinflammatory cytokines in the brain. Minocycline protects against LPS-induced elevation in proinflammatory cytokine transcripts and co-treatment with indomethacin plus minocycline had no effect on LPS-induced elevation in transcripts of proinflammatory cytokines in the brain. Thus, treatment with indomethacin could worsen neuroinflammation via increased iNOS both at gene and protein expression levels. In addition, minocycline’s anti-neuroinflammatory effects are annulled in the presence of indomethacin.

## Supplemental Information

10.7717/peerj.10391/supp-1 Supplemental Information 1 Relative expression of *Il10*, *Il1b*, *Tnf* and *Nos2* mRNA in the brain of control (vehicle-only ), LPS-inoculated vehicle-treated and LPS-inoculated indomethacin-treated at 4 h post LPS/vehicle inoculation.

10.7717/peerj.10391/supp-2 Supplemental Information 2 Expression of Iba-1 protein in the brain of control (vehicle-only) and LPS-inoculated mice at 4 h post LPS/vehicle inoculation.

10.7717/peerj.10391/supp-3 Supplemental Information 3 Relative expression of *Il10*, *Il1b*, *Tnf* and *Nos2* mRNA in the brain of control (vehicle-only ), LPS-inoculated vehicle-treated and LPS-inoculated indomethacin plus minocycline-treated at 4 h post LPS/vehicle inoculation. Relative expression of *Il10*, *Il1b*, *Tnf* and *Nos2* mRNA in the brain of control (vehicle-only ), LPS-inoculated vehicle-treated and LPS-inoculated indomethacin plus minocycline-treated at 4 h post LPS/vehicle inoculation.

10.7717/peerj.10391/supp-4 Supplemental Information 4 Relative expression of *Il1b*, *Tnf* and *Nos2* mRNA in the brain of control (vehicle-only) and LPS-inoculated mice at 4 h post LPS/vehicle inoculation.

10.7717/peerj.10391/supp-5 Supplemental Information 5 Expression of iNOS protein in the brain of control (vehicle-only), LPS-inoculated vehicle-treated and LPS-inoculated indomethacin-treated at 4 h post LPS/vehicle inoculation.

10.7717/peerj.10391/supp-6 Supplemental Information 6 Calculations for Figure 1.

10.7717/peerj.10391/supp-7 Supplemental Information 7 Relative expression of *Il10*, *Il1b*, *Tnf* and *Nos2* mRNA in the brain of control (vehicle-only ), LPS-inoculated vehicle-treated and LPS-inoculated minocycline-treated at 4 h post LPS/vehicle inoculation.

10.7717/peerj.10391/supp-8 Supplemental Information 8 Relative expression of *Il1b*, *Tnf* and *Nos2* mRNA in the brain of control (vehicle-only) and indomethacin-treated mice at 4 h post treatement.

10.7717/peerj.10391/supp-9 Supplemental Information 9 Virtual-blot like image similar to traditional results obtained from electropherograms for Figure 1. Lane 1 and 2 is for actin, lane 3 and 4 is for Iba-1

10.7717/peerj.10391/supp-10 Supplemental Information 10 Electropherograms of actin for Figure 1.

10.7717/peerj.10391/supp-11 Supplemental Information 11 Electropherograms of Iba-1for Figure 1.

10.7717/peerj.10391/supp-12 Supplemental Information 12 Illustration of Wes^™^ data analysis. Data analysis using ratio the between of the areas of the resulting electropherograms for Iba-1 and β-actin generated during the Wes^™^ capillary-based protein electrophoresis.

10.7717/peerj.10391/supp-13 Supplemental Information 13 Effect of inoculation of BALB/c mice with lipopolysaccharide (LPS) on Iba-1 protein expression. Protein expression of Iba-1 in the brain of control (vehicle-only) and LPS-inoculated mice at 4 h post LPS/vehicle inoculation. Each box and whiskers represents the median and interquartile range of the values obtained from five animals. Statistically significant difference with vehicle-treated control animals: **p < 0.01 (Mann–Whitney *U* test).

10.7717/peerj.10391/supp-14 Supplemental Information 14 Effect of inoculation of BALB/c mice with lipopolysaccharide (LPS) on *Il10*, *Il1b*, *Tnf* and *Nos2* mRNA expression. Relative expression of (A) *Il10*, (B) *Il1b*, (C) *Tnf* and (D) *Nos2* mRNA in the brain of control (vehicle-only) and LPS-inoculated mice at 4 h post LPS/vehicle inoculation. Each bar or box and whiskers represents the mean ± S.E.M (A and B) or median and interquartile range (C), respectively, of the values obtained from eight animals. Statistically significant difference with vehicle-treated control animals: ***p < 0.01 (Student’s *t* test for *Il10*, *Il1b* and *Tnf* or Mann–Whitney *U* test for *Nos2*).

10.7717/peerj.10391/supp-15 Supplemental Information 15 Supplemental results.

## Additional Information and Declarations

### Data Availability

The following information was supplied regarding data availability: The protein expression raw data and real-time PCR raw data are available in the Supplemental Files. The protein expression was done using the ProteinSimple’s WesTM Capillary-Based Protein Electrophoresis; thus, the raw data comes as electropherograms from which virtual-blot-like images similar to traditional results as well as a Supplemental File showing the height and area under the electropherograms are generated.

## References

[ref-1] Aeberhard EE, Henderson SA, Arabolos NS, Griscavage JM, Castro FE, Barrett CT, Ignarro LJJB, communications br (1995). Nonsteroidal anti-inflammatory drugs inhibit expression of the inducible nitric oxide synthase gene. Biochemical and Biophysical Research Communications.

[ref-2] Aid S, Langenbach R, Bosetti F (2008). Neuroinflammatory response to lipopolysaccharide is exacerbated in mice genetically deficient in cyclooxygenase-2. Journal of Neuroinflammation.

[ref-3] Aly E, Khajah MA, Masocha W (2020). β-Caryophyllene, a CB2-receptor-selective phytocannabinoid, suppresses mechanical allodynia in a mouse model of antiretroviral-induced neuropathic pain. Molecules.

[ref-4] Banks WA, Gray AM, Erickson MA, Salameh TS, Damodarasamy M, Sheibani N, Meabon JS, Wing EE, Morofuji Y, Cook DG, Reed MJ (2015). Lipopolysaccharide-induced blood-brain barrier disruption: roles of cyclooxygenase, oxidative stress, neuroinflammation, and elements of the neurovascular unit. Journal of Neuroinflammation.

[ref-5] Biesmans S, Meert TF, Bouwknecht JA, Acton PD, Davoodi N, De Haes P, Kuijlaars J, Langlois X, Matthews LJ, Ver Donck L, Hellings N, Nuydens R (2013). Systemic immune activation leads to neuroinflammation and sickness behavior in mice. Mediators of Inflammation.

[ref-6] Blais V, Turrin NP, Rivest S (2005). Cyclooxygenase 2 (COX-2) inhibition increases the inflammatory response in the brain during systemic immune stimuli. Journal of Neurochemistry.

[ref-7] Catorce M, Gevorkian G (2016). LPS-induced murine neuroinflammation model: main features and suitability for pre-clinical assessment of nutraceuticals. Current Neuropharmacology.

[ref-8] Chen WW, Zhang X, Huang WJ (2016). Role of neuroinflammation in neurodegenerative diseases (Review). Molecular Medicine Reports.

[ref-9] Conti P, Lauritano D, Caraffa A, Gallenga CE, Kritas SK, Ronconi G, Martinotti S (2020). Microglia and mast cells generate proinflammatory cytokines in the brain and worsen inflammatory state: suppressor effect of IL-37. European Journal of Pharmacology.

[ref-10] Czapski GA, Cakala M, Chalimoniuk M, Gajkowska B, Strosznajder JB (2007). Role of nitric oxide in the brain during lipopolysaccharide-evoked systemic inflammation. Journal of Neuroscience Research.

[ref-11] De Jong D, Jansen R, Hoefnagels W, Jellesma-Eggenkamp M, Verbeek M, Borm G, Kremer B (2008). No effect of one-year treatment with indomethacin on Alzheimer’s disease progression: a randomized controlled trial. PLOS ONE.

[ref-12] Di Girolamo G, Farina M, Riberio ML, Ogando D, Aisemberg J, De los Santos AR, Marti ML, Franchi AM (2003). Effects of cyclooxygenase inhibitor pretreatment on nitric oxide production, nNOS and iNOS expression in rat cerebellum. British Journal of Pharmacology.

[ref-13] Eccleston C, Cooper TE, Fisher E, Anderson B, Wilkinson NMR (2017). Non-steroidal anti-inflammatory drugs (NSAIDs) for chronic non-cancer pain in children and adolescents. Cochrane Database of Systematic Reviews.

[ref-14] Edan RA, Luqmani YA, Masocha W (2013). COL-3, a chemically modified tetracycline, inhibits lipopolysaccharide-induced microglia activation and cytokine expression in the brain. PLOS ONE.

[ref-15] Fan LW, Pang Y, Lin S, Rhodes PG, Cai Z (2005). Minocycline attenuates lipopolysaccharide-induced white matter injury in the neonatal rat brain. Neuroscience.

[ref-16] Giulian D, Baker TJ (1986). Characterization of ameboid microglia isolated from developing mammalian brain. Journal of Neuroscience.

[ref-17] Guo G, Bhat NR (2007). p38α MAP kinase mediates hypoxia-induced motor neuron cell death: a potential target of minocycline’s neuroprotective action. Neurochemical Research.

[ref-18] Guo ML, Buch S (2019). Neuroinflammation & pre-mature aging in the context of chronic HIV infection and drug abuse: role of dysregulated autophagy. Brain research.

[ref-19] Henry CJ, Huang Y, Wynne A, Hanke M, Himler J, Bailey MT, Sheridan JF, Godbout JP (2008). Minocycline attenuates lipopolysaccharide (LPS)-induced neuroinflammation, sickness behavior, and anhedonia. Journal of Neuroinflammation.

[ref-20] Hom GJ, Grant SK, Wolfe G, Bach TJ, MacIntyre DE, Hutchinson NI (1995). Lipopolysaccharide-induced hypotension and vascular hyporeactivity in the rat: tissue analysis of nitric oxide synthase mRNA and protein expression in the presence and absence of dexamethasone, NG-monomethyl-L-arginine or indomethacin. Journal of Pharmacology and Experimental Therapeutics.

[ref-21] Hoogland ICM, Houbolt C, Van Westerloo DJ, Van Gool WA, Van de Beek D (2015). Systemic inflammation and microglial activation: systematic review of animal experiments. Journal of Neuroinflammation.

[ref-22] Hou Y, Xie G, Liu X, Li G, Jia C, Xu J, Wang B (2016). Minocycline protects against lipopolysaccharide-induced cognitive impairment in mice. Psychopharmacology.

[ref-23] Hu JF, Song XY, Chu SF, Chen J, Ji HJ, Chen XY, Yuan YH, Han N, Zhang JT, Chen NH (2011). Inhibitory effect of ginsenoside Rg1 on lipopolysaccharide-induced microglial activation in mice. Brain Research.

[ref-24] Huang Y, Henry CJ, Dantzer R, Johnson RW, Godbout JP (2008). Exaggerated sickness behavior and brain proinflammatory cytokine expression in aged mice in response to intracerebroventricular lipopolysaccharide. Neurobiology of Aging.

[ref-25] Hussain M, Javeed A, Ashraf M, Al-Zaubai N, Stewart A, Mukhtar MM (2012). Non-steroidal anti-inflammatory drugs, tumour immunity and immunotherapy. Pharmacological Research.

[ref-26] Iliev A, Traykov V, Stoykov I, Yakimova K (2001). Estradiol inhibits astrocytic GFAP expression in an animal model of neuroinflammation. Methods and Findings in Experimental and Clinical Pharmacology.

[ref-27] Ito D, Imai Y, Ohsawa K, Nakajima K, Fukuuchi Y, Kohsaka S (1998). Microglia-specific localisation of a novel calcium binding protein, Iba1. Molecular Brain Research.

[ref-28] Ito D, Tanaka K, Suzuki S, Dembo T, Fukuuchi Y (2001). Enhanced expression of Iba1, ionized calcium-binding adapter molecule 1, after transient focal cerebral ischemia in rat brain. Stroke.

[ref-29] Jacobs RA, Satta MA, Dahia PLM, Chew SL, Grossman AB (1997). Induction of nitric oxide synthase and interleukin-1β, but not heme oxygenase, messenger RNA in rat brain following peripheral administration of endotoxin. Molecular Brain Research.

[ref-30] Kim S-S, Kong P-J, Kim B-S, Sheen D-H, Nam S-Y, Chun W (2004). Inhibitory action of minocycline on lipopolysaccharide-lnduced release of nitric oxide and prostaglandin E2 in BV2 microglial cells. Archives of Pharmacal Research.

[ref-31] Layé S, Parnet P, Goujon E, Dantzer R (1994). Peripheral administration of lipopolysaccharide induces the expression of cytokine transcripts in the brain and pituitary of mice. Molecular Brain Research.

[ref-32] Lee SM, Yune TY, Kim SJ, Kim YC, Oh YJ, Markelonis GJ, Oh TH (2004). Minocycline inhibits apoptotic cell death via attenuation of TNF-alpha expression following iNOS/NO induction by lipopolysaccharide in neuron/glia co-cultures. Journal of Neurochemistry.

[ref-33] Livak KJ, Schmittgen TD (2001). Analysis of relative gene expression data using real-time quantitative PCR and the 2−ΔΔCT method. Methods.

[ref-34] Loane DJ, Byrnes KR (2010). Role of microglia in neurotrauma. Neurotherapeutics.

[ref-35] Lull ME, Block ML (2010). Microglial activation and chronic neurodegeneration. Neurotherapeutics.

[ref-36] Marchalant Y, Rosi S, Wenk GL (2007). Anti-inflammatory property of the cannabinoid agonist WIN-55212-2 in a rodent model of chronic brain inflammation. Neuroscience.

[ref-37] Masocha W (2009). Systemic lipopolysaccharide (LPS)-induced microglial activation results in different temporal reduction of CD200 and CD200 receptor gene expression in the brain. Journal of Neuroimmunology.

[ref-38] Masocha W, Parvathy SS (2009). Assessment of weight bearing changes and pharmacological antinociception in mice with LPS-induced monoarthritis using the Catwalk gait analysis system. Life Sciences Part 1 Physiology & Pharmacology.

[ref-39] Meneses G, Cardenas G, Espinosa A, Rassy D, Perez-Osorio IN, Barcena B, Fleury A, Besedovsky H, Fragoso G, Sciutto E (2019). Sepsis: developing new alternatives to reduce neuroinflammation and attenuate brain injury. Annals of the New York Academy of Sciences.

[ref-40] Molteni M, Rossetti C (2017). Neurodegenerative diseases: the immunological perspective. Journal of Neuroimmunology.

[ref-41] Murray CL, Skelly DT, Cunningham C (2011). Exacerbation of CNS inflammation and neurodegeneration by systemic LPS treatment is independent of circulating IL-1β and IL-6. Journal of Neuroinflammation.

[ref-42] Na YR, Yoon YN, Son D, Jung D, Gu GJ, Seok SH (2015). Consistent inhibition of cyclooxygenase drives macrophages towards the inflammatory phenotype. PLOS ONE.

[ref-43] Nakamori T, Morimoto A, Yamaguchi K, Watanabe T, Murakami N (1994). Interleukin-1 beta production in the rabbit brain during endotoxin-induced fever. Journal of Physiology.

[ref-44] Nalamachu S, Wortmann R (2014). Role of indomethacin in acute pain and inflammation management: a review of the literature. Postgraduate Medicine.

[ref-45] Neupane S, Srivastav S, Bhurtel S, Katila N, Shadfar S, Park PH, Hong JT, Choi DY (2018). Enhanced neuroinflammatory responses after systemic LPS injection in IL-32β transgenic mice. Journal of Chemical Neuroanatomy.

[ref-46] Pang L, Hoult JR (1996). Induction of cyclooxygenase and nitric oxide synthase in endotoxin-activated J774 macrophages is differentially regulated by indomethacin: enhanced cyclooxygenase-2 protein expression but reduction of inducible nitric oxide synthase. European Journal of Pharmacology.

[ref-47] Plane JM, Shen Y, Pleasure DE, Deng W (2010). Prospects for minocycline neuroprotection. Archives of Neurology.

[ref-48] Prechel MM, Ding C, Washington RL, Kolodziej MS, Young MR (2000). In vivo indomethacin treatment causes microgial activation in adult mice. Neurochemical Research.

[ref-49] Qin L, Wu X, Block ML, Liu Y, Breese GR, Hong JS, Knapp DJ, Crews FT (2007). Systemic LPS causes chronic neuroinflammation and progressive neurodegeneration. Glia.

[ref-50] Reinert KRS, Umphlet CD, Quattlebaum A, Boger HA (2014). Short-term effects of an endotoxin on substantia nigra dopamine neurons. Brain Research.

[ref-51] Rogers J, Kirby LC, Hempelman SR, Berry DL, McGeer PL, Kaszniak AW, Zalinski J, Cofield M, Mansukhani L, Willson P, Kogan F (1993). Clinical trial of indomethacin in Alzheimer’s disease. Neurology.

[ref-52] Sacco S, Agnello D, Sottocorno M, Lozza G, Monopoli A, Villa P, Ghezzi P (1998). Nonsteroidal anti-inflammatory drugs increase tumor necrosis factor production in the periphery but not in the central nervous system in mice and rats. Journal of Neurochemistry.

[ref-53] Sawada M, Kondo N, Suzumura A, Marunouchi T (1989). Production of tumor necrosis factor-alpha by microglia and astrocytes in culture. Brain Research.

[ref-54] Schain M, Kreisl WC (2017). Neuroinflammation in neurodegenerative disorders—a review. Current Neurology and Neuroscience Reports.

[ref-55] Smith JA, Das A, Ray SK, Banik NL (2012). Role of pro-inflammatory cytokines released from microglia in neurodegenerative diseases. Brain Research Bulletin.

[ref-56] Stirling DP, Koochesfahani KM, Steeves JD, Tetzlaff W (2005). Minocycline as a neuroprotective agent. Neuroscientist.

[ref-57] Szot P, Franklin A, Figlewicz DP, Beuca TP, Bullock K, Hansen K, Banks WA, Raskind MA, Peskind ER (2017). Multiple lipopolysaccharide (LPS) injections alter interleukin 6 (IL-6), IL-7, IL-10 and IL-6 and IL-7 receptor mRNA in CNS and spleen. Neuroscience.

[ref-58] Teeling JL, Felton LM, Deacon RMJ, Cunningham C, Rawlins JNP, Perry VH (2007). Sub-pyrogenic systemic inflammation impacts on brain and behavior, independent of cytokines. Brain, Behavior, and Immunity.

[ref-59] Tikka TM, Vartiainen NE, Goldsteins G, Oja SS, Andersen PM, Marklund SL, Koistinaho J (2002). Minocycline prevents neurotoxicity induced by cerebrospinal fluid from patients with motor neurone disease. Brain.

[ref-60] Tomás-Camardiel M, Rite I, Herrera A, De Pablos R, Cano J, Machado A, Venero J (2004). Minocycline reduces the lipopolysaccharide-induced inflammatory reaction, peroxynitrite-mediated nitration of proteins, disruption of the blood–brain barrier, and damage in the nigral dopaminergic system. Neurobiology of Disease.

[ref-61] Voronova IP, Khramova GM, Kulikova EA, Petrovskii DV, Bazovkina DV, Kulikov AV (2016). 5-HT2A receptors control body temperature in mice during LPS-induced inflammation via regulation of NO production. Pharmacological Research.

[ref-62] Wolf SA, Boddeke HW, Kettenmann H (2017). Microglia in physiology and disease. Annual Review of Physiology.

[ref-63] Xu L, He D, Bai Y (2016). Microglia-mediated inflammation and neurodegenerative disease. Molecular Neurobiology.

[ref-64] Yang L, Zhou R, Tong Y, Chen P, Shen Y, Miao S, Liu X (2020). Neuroprotection by dihydrotestosterone in LPS-induced neuroinflammation. Neurobiology of Disease.

[ref-65] Yrjänheikki J, Keinänen R, Pellikka M, Hökfelt T, Koistinaho J (1998). Tetracyclines inhibit microglial activation and are neuroprotective in global brain ischemia. Proceedings of the National Academy of Sciences of the United States of America.

