# Supplementary material for: Indomethacin augments lipopolysaccharide-induced expression of inflammatory molecules in the mouse brain
Source: PeerJ. 2020 Nov 18;8:e10391. doi: 10.7717/peerj.10391 (PMC7680052; doi:10.7717/peerj.10391)
Supplement: Supplemental Information 1 [file peerj-08-10391-s001.docx]

**Relative expression of *Il10*, *Il1b*, *Tnf* and *Nos2* mRNA in the brain of control (vehicle-only****), LPS-inoculated vehicle-treated and LPS-inoculated indomethacin-treated at 4 h post LPS/vehicle inoculation.**

| **Animal number** | **Gene** | | | | | | | | |  |  |  |
| --- | --- | --- | --- | --- | --- | --- | --- | --- | --- | --- | --- | --- |
|  | ***Il1b*** | | | ***Tnf*** | | | ***Nos2*** | | | ***Il10*** | | |
|  | **Control*** | **LPS^#^** | **LPS + Indo^$^** | **Control*** | **LPS^#^** | **LPS + Indo^$^** | **Control*** | **LPS^#^** | **LPS + Indo^$^** | **Control*** | **LPS^#^** | **LPS + Indo^$^** |
| 1 | 0.901193 | 2.517936 | 39.69013 | 0.894968 | 2.72126 | 22.83606 | 0.928603 | 1.741624 | 6.939668 | 0.821500 | 1.177886 | 5.734628 |
| 2 | 1.169432 | 2.89128 | 9.1941 | 0.869048 | 2.974351 | 18.76764 | 0.737203 | 3.864013 | 5.668106 | 1.286707 | 1.835322 | 1.727936 |
| 3 | 1.440472 | 7.455517 | 5.189956 | 1.321804 | 4.484483 | 6.293203 | 1.290741 | 4.336507 | 1.642008 | 0.961961 | 0.734924 | 1.091466 |
| 4 | 0.658722 | 10.50042 | 23.58268 | 0.972706 | 11.40616 | 41.67358 | 1.131733 | 4.521809 | 8.782172 | 1.155433 | 1.536236 | 2.302073 |
| 5 | 1.096411 | 8.049443 | 9.70496 | 1.159842 | 10.69646 | 13.463 | 0.936691 | 2.446712 | 3.836022 | 0.851159 | 3.478584 | 1.375923 |
| 6 | 0.774313 | 4.741759 | 8.462138 | 0.992297 | 5.557597 | 11.60489 | 1.344756 | 2.398963 | 4.371145 |  |  |  |
| 7 | 1.177904 | 3.831269 | 3.155107 | 0.868879 | 5.474755 | 2.700097 | 0.79389 | 3.619886 | 1.266517 |  |  |  |
| 8 | 1.837132 | 6.490167 | 6.206969 | 1.591674 | 6.128544 | 6.01561 | 1.099235 | 3.597181 | 3.949035 |  |  |  |
| 9 | 0.703142 | 4.082696 | 103.8115 | 0.670733 | 2.903 | 70.44593 | 0.83946 | 1.462733 | 15.79212 |  |  |  |
| 10 | 0.774134 | 9.349282 | 184.8429 | 0.936691 | 7.708113 | 116.2009 | 1.083701 | 4.409148 | 17.89974 |  |  |  |

*Control (vehicles only- injected) mice

^#^ LPS-inoculated vehicle-treated

^$^ LPS-inoculated indomethacin-treated
