# Supplementary material for: Indomethacin augments lipopolysaccharide-induced expression of inflammatory molecules in the mouse brain
Source: PeerJ. 2020 Nov 18;8:e10391. doi: 10.7717/peerj.10391 (PMC7680052; doi:10.7717/peerj.10391)
Supplement: Supplemental Information 2 [file peerj-08-10391-s002.docx]

**Expression of Iba-1 protein in the brain of control (vehicle-only) and LPS-inoculated mice at 4 h post LPS/vehicle inoculation.**

| **Animal number** | **Control*** | **LPS^#^** |
| --- | --- | --- |
| 1 | 0.382727 | 0.710151 |
| 2 | 0.484948 | 0.730713 |
| 3 | 0.530068 | 1.152195 |
| 4 | 0.614525 | 0.896707 |
| 5 | 0.611915 | 0.748797 |

*Control (vehicles only- injected) mice

^#^ LPS-inoculated vehicle-treated
