# Supplementary material for: Indomethacin augments lipopolysaccharide-induced expression of inflammatory molecules in the mouse brain
Source: PeerJ. 2020 Nov 18;8:e10391. doi: 10.7717/peerj.10391 (PMC7680052; doi:10.7717/peerj.10391)
Supplement: Supplemental Information 3 — Relative expression of Il10, Il1b, Tnf and Nos2 mRNA in the brain of control (vehicle-only ), LPS-inoculated vehicle-treated and LPS-inoculated indomethacin plus minocycline-treated at 4 h post LPS/vehicle inoculation. [file peerj-08-10391-s003.docx]

**Relative expression of *Il10*, *Il1b*, *Tnf* and *Nos2* mRNA in the brain of control (vehicle-only****), LPS-inoculated vehicle-treated and LPS-inoculated indomethacin plus minocycline-treated at 4 h post LPS/vehicle inoculation.**

| **Animal number** | **Gene** | | | | | | | | |
| --- | --- | --- | --- | --- | --- | --- | --- | --- | --- |
|  | ***Il1b*** | | | ***Tnf*** | | | ***Nos2*** | | |
|  | **Control*** | **LPS^#^** | **LPS + IPM^$^** | **Control*** | **LPS^#^** | **LPS + IPM ^$^** | **Control*** | **Mino ^#^** | **LPS + IPM^$^** |
| 1 | 1.225086 | 1.456376 | 4.201633 | 1.376845 | 11.434860 | 11.759600 | 1.220533 | 9.497258 | 8.673681 |
| 2 | 1.003553 | 5.217227 | 6.133384 | 1.127140 | 16.326380 | 15.773890 | 0.825388 | 21.514600 | 15.518430 |
| 3 | 0.813379 | 2.501714 | 6.165210 | 0.644372 | 4.994014 | 47.868340 | 0.992641 | 4.083514 | 41.240150 |
| 4 | 0.924400 | 5.422866 | 4.463215 | 0.907051 | 5.985294 | 11.108300 | 1.907060 | 2.573889 | 12.043460 |
| 5 | 1.223761 | 20.765100 | 6.507409 | 0.755999 | 18.053690 | 17.852240 | 0.531761 | 2.511269 | 13.701020 |
| 6 | 0.883982 | 21.235250 | 12.188280 | 1.458301 | 18.946010 | 11.508190 | 0.986096 | 6.091581 | 4.967374 |
| 7 | 0.977657 | 9.331226 | 53.031910 | 1.842609 | 7.983105 | 33.955080 | 0.895060 | 5.119095 | 7.247332 |
| 8 | 1.244127 | 6.221087 | 3.754157 | 1.104288 | 4.417765 | 3.628958 | 0.859790 | 3.743071 | 1.199320 |
| 9 | 0.822146 | 8.648001 | 2.366228 | 0.491456 | 6.925332 | 2.967515 | 1.299439 | 6.217447 | 1.362195 |
| 10 |  |  | 7.535947 |  |  | 7.803144 |  |  | 7.518179 |
| 11 |  |  | 3.168134 |  |  | 3.480955 |  |  | 2.655633 |
| 12 |  |  | 4.523481 |  |  | 6.216417 |  |  | 6.188496 |
| 13 |  |  | 175.788900 |  |  | 118.590900 |  |  | 21.765770 |
| 14 |  |  | 385.458900 |  |  | 153.541300 |  |  | 45.272260 |

*Control (vehicles only- injected) mice

^#^ LPS-inoculated vehicle-treated

^$^ LPS-inoculated indomethacin plus minocycline-treated
