# Supplementary material for: Indomethacin augments lipopolysaccharide-induced expression of inflammatory molecules in the mouse brain
Source: PeerJ. 2020 Nov 18;8:e10391. doi: 10.7717/peerj.10391 (PMC7680052; doi:10.7717/peerj.10391)
Supplement: Supplemental Information 4 [file peerj-08-10391-s004.docx]

**Relative expression of *Il1b*, *Tnf* and** ***Nos2* mRNA in the brain of control (vehicle-only) and LPS-inoculated mice at 4 h post LPS/vehicle inoculation.**

| **Animal number** | **Gene** | | | | | |  |  |
| --- | --- | --- | --- | --- | --- | --- | --- | --- |
|  | ***Il1b*** | | ***Tnf*** | | ***Nos2*** | | ***Il10*** |  |
|  | **Control*** | **LPS^#^** | **Control*** | **Control*** | **Control*** | **LPS^#^** | **Control*** | **LPS^#^** |
| 1 | 0.8870398 | 47.080930 | 1.222640 | 17.535420 | 0.7855024 | 3.439076 | 5.233798 | 5.068323 |
| 2 | 1.208628 | 43.750800 | 1.428829 | 22.388200 | 1.195202 | 1.943227 | 0.221533 | 2.195691 |
| 3 | 0.9547561 | 32.729150 | 0.7348899 | 16.350540 | 0.9925563 | 2.027011 | 4.649283 | 0.634698 |
| 4 | 0.9769483 | 43.435540 | 0.7789306 | 14.777530 | 1.073139 | 1.479546 | 0.409067 | 1.920890 |
| 5 | 0.8773149 | 27.170450 | 0.8800302 | 21.011270 | 1.018927 | 2.600910 | 0.540104 | 4.121040 |
| 6 | 1.196373 | 30.885690 | 0.7762671 | 15.332540 | 0.907006 | 3.921621 | 1.326454 | 2.521313 |
| 7 | 1.203414 | 71.265570 | 1.278890 | 26.062280 | 1.050457 | 13.093240 | 1.797188 | 1.101173 |
| 8 | 0.7917042 | 23.983440 | 1.144612 | 12.222140 | 1.030075 | 3.664839 | 0.352210 | 0.499143 |

*Control (vehicles only- injected) mice

^#^ LPS-inoculated vehicle-treated
