# Supplementary material for: Indomethacin augments lipopolysaccharide-induced expression of inflammatory molecules in the mouse brain
Source: PeerJ. 2020 Nov 18;8:e10391. doi: 10.7717/peerj.10391 (PMC7680052; doi:10.7717/peerj.10391)
Supplement: Supplemental Information 5 [file peerj-08-10391-s005.docx]

**Expression of iNOS protein in the brain of control (vehicle-only), LPS-inoculated vehicle-treated and LPS-inoculated indomethacin-treated at 4 h post LPS/vehicle inoculation.**

| **Animal number** | **Control*** | **LPS^#^** | **LPS + Indo^$^** |
| --- | --- | --- | --- |
| 1 | 4181.329000 | 6751.979000 | 9522.610000 |
| 2 | 3768.905000 | 8619.905000 | 7969.002000 |
| 3 | 5198.739000 | 6765.065000 | 7212.264000 |
| 4 | 10478.720000 | 7957.931000 | 5631.112000 |
| 5 | 3131.281000 | 5149.926000 | 11235.280000 |
| 6 | 4245.160000 | 5728.406000 | 7334.265000 |
| 7 | 4789.340000 | 7788.820000 |  |
| 8 | 4898.740000 | 7355.256000 |  |

*Control (vehicles only- injected) mice

^#^ LPS-inoculated vehicle-treated

^$^ LPS-inoculated indomethacin-treated
