# Supplementary material for: Indomethacin augments lipopolysaccharide-induced expression of inflammatory molecules in the mouse brain
Source: PeerJ. 2020 Nov 18;8:e10391. doi: 10.7717/peerj.10391 (PMC7680052; doi:10.7717/peerj.10391)
Supplement: Supplemental Information 7 [file peerj-08-10391-s007.docx]

**Relative expression of *Il10*, *Il1b*, *Tnf* and *Nos2* mRNA in the brain of control (vehicle-only****), LPS-inoculated vehicle-treated and LPS-inoculated minocycline-treated at 4 h post LPS/vehicle inoculation.**

| **Animal number** | **Gene** | | | | | | | | |
| --- | --- | --- | --- | --- | --- | --- | --- | --- | --- |
|  | ***Il1b*** | | | ***Tnf*** | | | ***Nos2*** | | |
|  | **Control*** | **LPS^#^** | **LPS + Mino^$^** | **Control*** | **LPS^#^** | **LPS + Mino ^$^** | **Control*** | **Mino ^#^** | **LPS + Indo^$^** |
| 1 | 0.8870398 | 47.080930 | 20.321540 | 1.222640 | 17.535420 | 6.720336 | 0.7855024 | 3.439076 | 6.733333 |
| 2 | 1.208628 | 43.750800 | 9.312300 | 1.428829 | 22.388200 | 8.534264 | 1.195202 | 1.943227 | 2.940825 |
| 3 | 0.9547561 | 32.729150 | 7.785938 | 0.7348899 | 16.350540 | 4.720479 | 0.9925563 | 2.027011 | 2.423156 |
| 4 | 0.9769483 | 43.435540 | 14.934440 | 0.7789306 | 14.777530 | 9.029211 | 1.073139 | 1.479546 | 4.411861 |
| 5 | 0.8773149 | 27.170450 | 3.229476 | 0.8800302 | 21.011270 | 2.720852 | 1.018927 | 2.600910 | 2.479559 |
| 6 | 1.196373 | 30.885690 | 9.948415 | 0.7762671 | 15.332540 | 6.570144 | 0.907006 | 3.921621 | 4.528657 |
| 7 | 1.203414 | 71.265570 | 3.742926 | 1.278890 | 26.062280 | 2.424563 | 1.050457 | 13.093240 | 1.847597 |
| 8 | 0.7917042 | 23.983440 | 10.062610 | 1.144612 | 12.222140 | 6.310226 | 1.030075 | 3.664839 | 7.638468 |

*Control (vehicles only- injected) mice

^#^ LPS-inoculated vehicle-treated

^$^ LPS-inoculated minocycline-treated
