# Supplementary material for: Indomethacin augments lipopolysaccharide-induced expression of inflammatory molecules in the mouse brain
Source: PeerJ. 2020 Nov 18;8:e10391. doi: 10.7717/peerj.10391 (PMC7680052; doi:10.7717/peerj.10391)
Supplement: Supplemental Information 8 [file peerj-08-10391-s008.docx]

**Relative expression of *Il1b*, *Tnf* and** ***Nos2* mRNA in the brain of control (vehicle-only) and indomethacin treated mice at 4 h post treatment.**

| **Animal number** | **Gene** | | | | | |  |  |
| --- | --- | --- | --- | --- | --- | --- | --- | --- |
|  | ***Il1b*** | | ***Tnf*** | | ***Nos2*** | | ***Il10*** |  |
|  | **Control*** | **INDO^#^** | **Control*** | **INDO^#^** | **Control*** | **INDO^#^** | **Control*** | **INDO^#^** |
| 1 | 0.898812 | 1.869076 | 1.329039 | 0.741825 | 0.718184 | 0.856856 | 1.540074 | 0.290089 |
| 2 | 1.429646 | 3.115128 | 0.766496 | 1.217721 | 1.078798 | 1.400710 | 0.969693 | 0.216474 |
| 3 | 0.960767 | 1.202768 | 0.830499 | 0.723442 | 1.118572 | 1.290764 | 1.110288 | 0.768100 |
| 4 | 0.809999 | 2.669109 | 1.181989 | 1.058203 | 1.153879 | 1.932933 | 0.603099 | 0.644127 |

*Control (vehicles only- injected) mice

^#^ Indomethacin-treated
