# Supplementary figures and images for: Indomethacin augments lipopolysaccharide-induced expression of inflammatory molecules in the mouse brain
Source: PeerJ. 2020 Nov 18;8:e10391. doi: 10.7717/peerj.10391 (PMC7680052; doi:10.7717/peerj.10391)

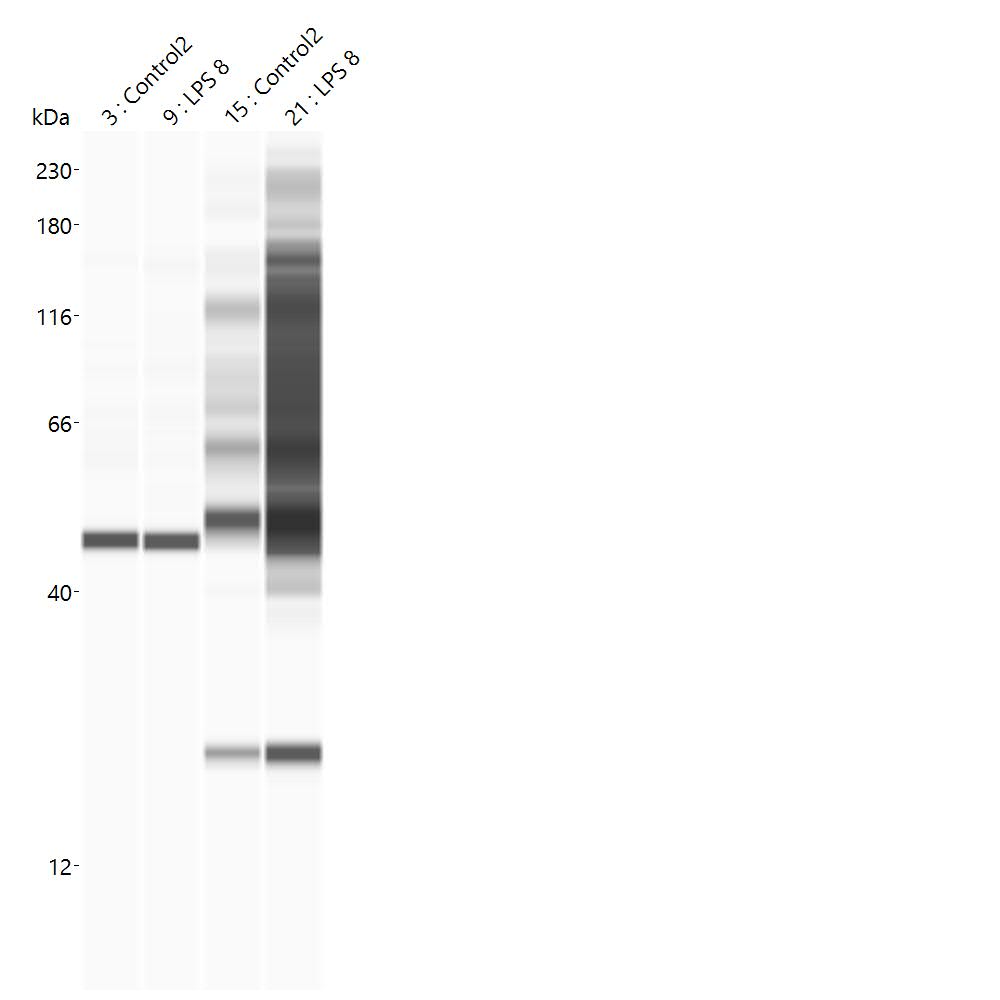

Supplement: Supplemental Information 9 — Lane 1 and 2 is for actin, lane 3 and 4 is for Iba-1 [file peerj-08-10391-s009.jpg]

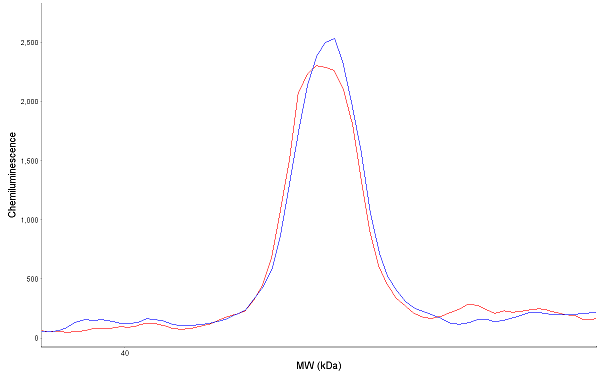

Supplement: Supplemental Information 10 [file peerj-08-10391-s010.png]

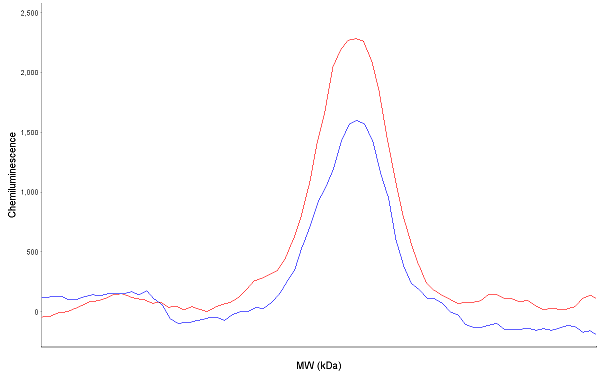

Supplement: Supplemental Information 11 [file peerj-08-10391-s011.png]

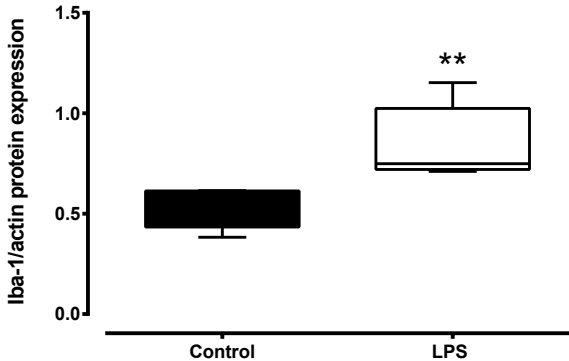

Supplement: Supplemental Information 13 — Protein expression of Iba-1 in the brain of control (vehicle-only) and LPS-inoculated mice at 4 h post LPS/vehicle inoculation. Each box and whiskers represents the median and interquartile range of the values obtained from five animals. Statistically significant difference with vehicle-treated control animals: **p < 0.01 (Mann–Whitney U test). [file peerj-08-10391-s013.pdf]

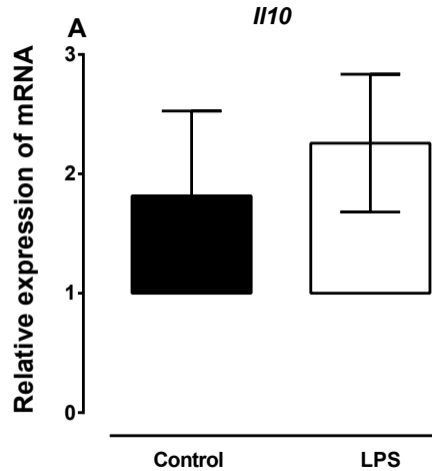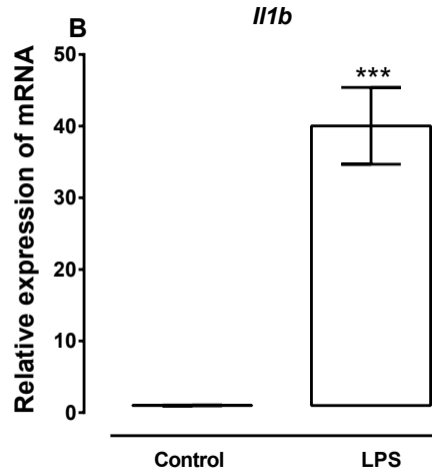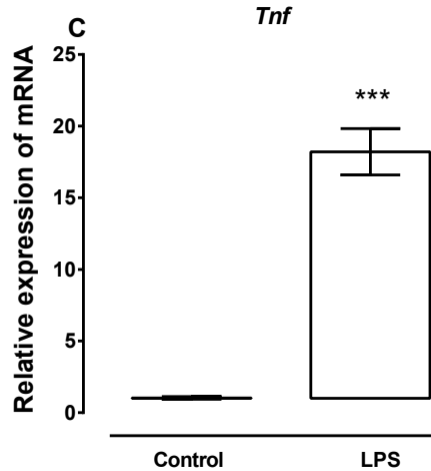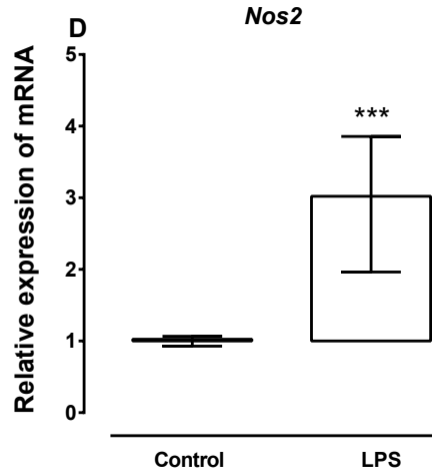

Supplement: Supplemental Information 14 — Relative expression of (A) Il10, (B) Il1b, (C) Tnf and (D) Nos2 mRNA in the brain of control (vehicle-only) and LPS-inoculated mice at 4 h post LPS/vehicle inoculation. Each bar or box and whiskers represents the mean ± S.E.M (A and B) or median and interquartile range (C), respectively, of the values obtained from eight animals. Statistically significant difference with vehicle-treated control animals: ***p < 0.01 (Student’s t test for Il10, Il1b and Tnf or Mann–Whitney U test for Nos2). [file peerj-08-10391-s014.pdf]
