# Supplementary material for: Indomethacin augments lipopolysaccharide-induced expression of inflammatory molecules in the mouse brain
Source: PeerJ. 2020 Nov 18;8:e10391. doi: 10.7717/peerj.10391 (PMC7680052; doi:10.7717/peerj.10391)
Supplement: Supplemental Information 12 — Data analysis using ratio the between of the areas of the resulting electropherograms for Iba-1 and β-actin generated during the Wes™ capillary-based protein electrophoresis. [file peerj-08-10391-s012.pdf]

Control LPS

Iba-1

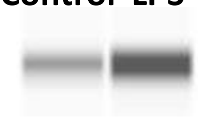

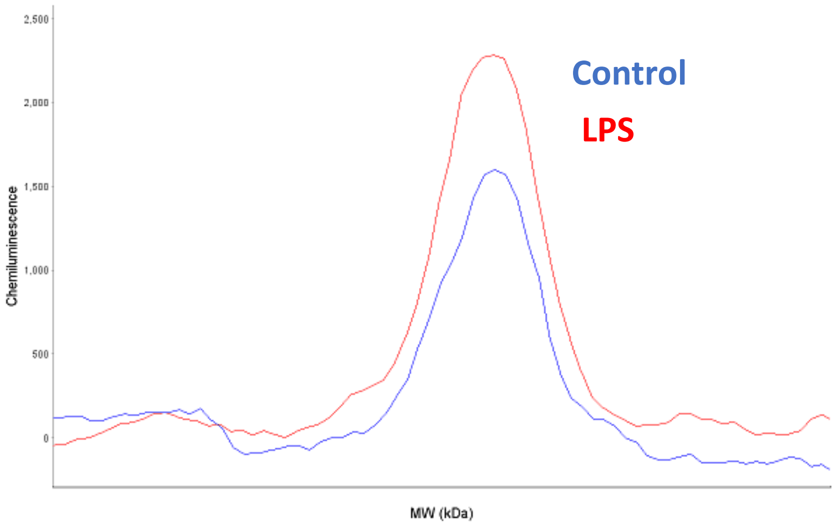

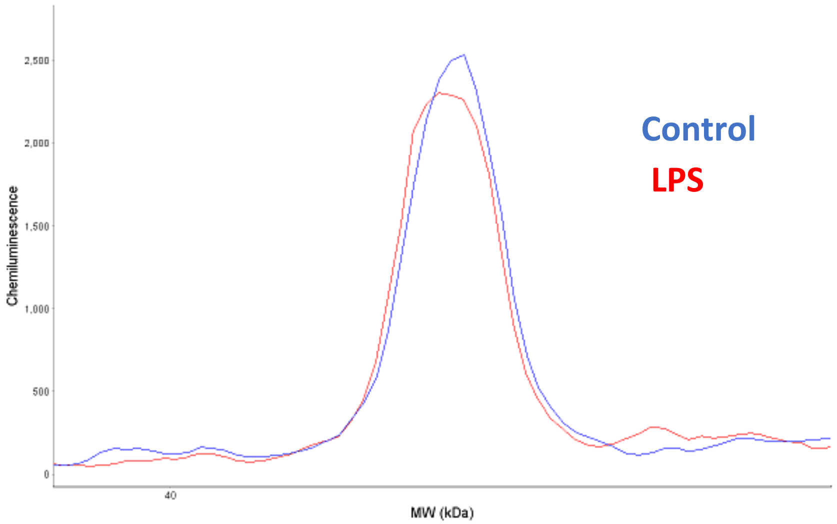

Actin

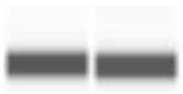

Sample	Primary	Height	Area
Control	Actin	2520.3	24278
LPS	Actin	2406.1	23871

Sample	Primary	Height	Area
Control	Iba-1	1606.3	16705
LPS	Iba-1	2354.5	26804

Sample			Iba-1/Actin
Control			0.68807151
LPS			1.12286875
