# Supplementary material for: Indomethacin augments lipopolysaccharide-induced expression of inflammatory molecules in the mouse brain
Source: PeerJ. 2020 Nov 18;8:e10391. doi: 10.7717/peerj.10391 (PMC7680052; doi:10.7717/peerj.10391)
Supplement: Supplemental Information 15 [file peerj-08-10391-s015.docx]

**Supplemental Material**

**Effect of LPS on Iba-1protein expression**

LPS induced the expression of the microglia marker Iba-1. The Mann–Whitney U test showed that the brains of LPS-inoculated mice had significantly upregulated Iba-1 protein expression after 4 hours of LPS injection compared to vehicle-treated group (Supplemental Figure 2; U = 0, p = 0.0079).

**Effect of LPS on Il10, Il1b, Tnf and Nos2 mRNA expression**

LPS inoculation increased *Il1b, Tnf* and *Nos2* mRNA, but had no effect on *Il10* mRNA, in the brains of mice compared to vehicle treatment. The unpaired Student’s *t* test showed there was no difference in the mRNA of *Il10* between the brains of mice inoculated with LPS compared to vehicle-treated control mice (t(14) = 0.4827, p = 0.6368; Supplemental Figure 3A). The unpaired Student’s *t* test showed that the mRNA of the cytokines genes *Il1b* and *Tnf* were significantly upregulated in the brains of mice inoculated with LPS compared to vehicle-treated control mice [t(14) = 7.277, p < 0.0001 for *Il1b*; t(14) =10.59, p < 0.0001 for *Tnf*; Supplemental Figure 3B and C]. The Mann–Whitney U test showed that the mRNA of *Nos2* was significantly upregulated in the brains of mice inoculated with LPS compared to vehicle-treated control mice (U = 0, p = 0.0002; Supplemental Figure 3D).
